# Seroprevalence of Herpes Simplex Virus 1 and 2 in a Population-Based Cohort in Japan

**DOI:** 10.2188/jea.JE20080061

**Published:** 2009-03-19

**Authors:** Yasufumi Doi, Toshiharu Ninomiya, Jun Hata, Koji Yonemoto, Yumihiro Tanizaki, Hisatomi Arima, Ying Liu, Mahbubur Rahman, Misuo Iida, Yutaka Kiyohara

**Affiliations:** 1 Department of Medicine and Clinical Science, Graduate School of Medical Sciences, Kyushu University, Fukuoka, Japan; 2 Department of Environmental Medicine, Graduate School of Medical Sciences, Kyushu University, Fukuoka, Japan; 3 Worldwide Epidemiology, GlaxoSmithKline R&D, Tokyo, Japan; 4 University of Texas Medical Branch, Galveston, Texas, USA

**Keywords:** herpes simplex virus types 1 and 2, seroprevalence, risk factors, Japan

## Abstract

**Background:**

There have been few population-based studies of the seroprevalence and correlates of herpes simplex virus type 1 (HSV-1) and type 2 (HSV-2) in Japan.

**Methods:**

We enrolled a total of 1244 adults, aged 18–59 years, from a population-based cohort in southern Japan, and tested their serum samples using an ELISA kit containing HSV type-specific antigens to glycoproteins G1 and G2.

**Results:**

The seroprevalence of HSV-1 and HSV-2 was 55.4% and 7.4% in men and 63.3% and 9.3% in women, respectively. Overall, 4 percent of the participants (2.3% of men and 5.0% of women) were co-infected with HSV-1 and HSV-2. The seroprevalences of both HSV-1 and HSV-2 increased with age in both sexes, and were always higher among women than among men in each age bracket. The prevalence of HSV-2 infection among HSV-1 infected individuals was lower than that among uninfected individuals, both in men and women. Male current drinkers, and male and female current smokers, were more likely to be infected with HSV-1 and HSV-2, as compared to never drinkers and never smokers, respectively.

**Conclusion:**

It is hoped that the estimates produced in this study will help in understanding the burden of these infections in Japan.

## INTRODUCTION

Herpes simplex virus (HSV) type 1 and type 2 infections are among the most common infections worldwide,^1^ although seroprevalence varies widely by country, region within individual countries, and population subgroup.^2^ Genital herpes, which is commonly attributed to HSV-2 infection and—in some developed countries—to HSV-1 infection, as well,^3^^–^^6^ is a significant public health concern and an important cause of psychological morbidity.^7^^,^^8^ Moreover, infection with HSV-1 and HSV-2 can cause serious conditions, including blindness, encephalitis, and neonatal infections; HSV-1 infection can also result in less serious conditions, such as orolabial and facial lesions. Thus, reliable prevalence estimates of these infections are needed to provide an epidemiological measure of the population burden.

While the epidemiology of HSV-1 and HSV-2 has been well studied in other industrialized countries, it has been less rigorously investigated in Japan. Two studies reported the seroprevalence of HSV-1 and HSV-2 in Japan,^9^^,^^10^ but they were based on small subgroups and were therefore not representative of the general population. Moreover, both of these studies were based on data generated during the 1980s and early 1990s. The objective of the current study was to estimate the age- and sex-specific seroprevalences of HSV-1 and HSV-2, along with their correlates, in a general Japanese population.

## METHODS

### Study design

A population-based prospective study of cardiovascular disease has been under way since 1961 in the town of Hisayama, a suburb of the Fukuoka metropolitan area on the island of Kyushu in Japan. The population of the town is approximately 8000 and has remained stable for 40 years. Based on data from the national census, the age and occupational distributions for Hisayama have been almost identical to those of Japan as a whole from 1961 to the present. Moreover, the nutritional intake for this sample is almost identical to that reported in the Japanese national nutrition survey.

In 2002, a total of 3328 participants aged at least 40 years consented to participate in this screening examination (participation rate: 77.6%). At that time, a venous blood sample was drawn from each subject and a serum repository was established so that the laboratory tests could be performed at a later date. Among the participants aged 40 to 59 years, 800 randomly selected men and women were included in the present study. To ensure that the sample was representative of the total adult population of the area, we also included all participants younger than 40 years, including 130 samples from individuals aged 18 to 29 years and 306 samples from those aged 30 to 39 years, after obtaining informed consent. Demographic data and information on several lifestyle variables were also recorded for all participants. The enrollment procedures for the study were reviewed and approved by the Kyushu University Institutional Review Board, and all participants gave written informed consent.

### Laboratory methods

Serum samples were kept frozen at −80 °C until tested. HSV-1 and HSV-2 type-specific antibody assays were performed at SRL Inc. (Tokyo, Japan) using a commercially available ELISA IgG assay kit containing the HSV type-specific antigens to glycoproteins G1 and G2 (HerpeSelect, Focus Diagnostics Inc., Cypress, CA). The antibody response to HSV glycoprotein G has been shown to be entirely type-specific. Use of type-specific proteins—glycoproteins G1 and G2—as antigens in immunologic assays now allows differentiation of previous infection with HSV-1, HSV-2, or both. The sensitivity and specificity of these tests have been previously evaluated and found satisfactory,^11^ and the tests have been commercially available since the late 1990s.^12^ Because of the extensive cross-antigenicity between the proteins of HSV-1 and HSV-2, no conventional serological method, other than assays with glycoproteins G1 and G2, has been proven capable of discriminating the antibodies of these 2 HSV types.^13^ To validate the assay procedure in the present study, 60 samples (5% of all samples) were selected randomly and retested. Fifty-nine samples (98.3%) for HSV-1 and 60 samples (100%) for HSV-2 showed concordance with the results obtained at the first testing. The discordant result was excluded from the final analyses. All samples were anonymous and were linked to patient demographic and lifestyle variables via a unique identification number.

### Statistical analysis

Univariate comparisons between different age groups were performed using the chi-square test. Age-and multivariate-adjusted logistic regression analyses were conducted to evaluate the association between the seroprevalence of HSV-1 and marital, smoking, and drinking statuses. A similar analysis was performed for HSV-2 infection. Odds ratios (ORs) with 95% confidence intervals (CIs) were estimated, and *P* < 0.05 was considered statistically significant in all analyses. The SAS software package version 8.2 (SAS Institute Inc., Cary, NC) was used to perform all statistical analyses.

## RESULTS

### Sociodemographic characteristics

Table 1 shows the sociodemographic status and comorbidities for men and women separately. Of the 1244 persons analyzed, 574 were men and 670 were women, with a mean age of 44 years and 43 years, respectively. Most of the men and women were married. Among men, 55.7% were current smokers and 75.1% were current drinkers; among women, the figures were 13.9% and 40.1%, respectively.

**Table 1.** Sociodemographic characteristics, comorbidities, and seroprevalences of HSV-1 and HSV-2 in men and women in Hisayama, Japan

	Men (*n* = 574)	Women (*n* = 670)
Age (years, mean ± SD)	44 ± 10	43 ± 11
Marital status (%)		
​ Married	86.8	84.4
​ Single	13.2	15.6
Smoking status (%)		
​ Never	22.8	81.0
​ Past	21.4	5.1
​ Current	55.8	13.9
Alcohol intake (%)		
​ Never	20.7	54.6
​ Past	4.0	3.7
​ Current	75.3	41.6
HSV-1 seroprevalence (%)	55.4	63.3
HSV-2 seroprevalence (%)	7.4	9.3
HSV-1 and HSV-2 coinfection (%)	2.3	5.0

### Seroprevalence of HSV-1 and HSV-2

**Figure 1.**
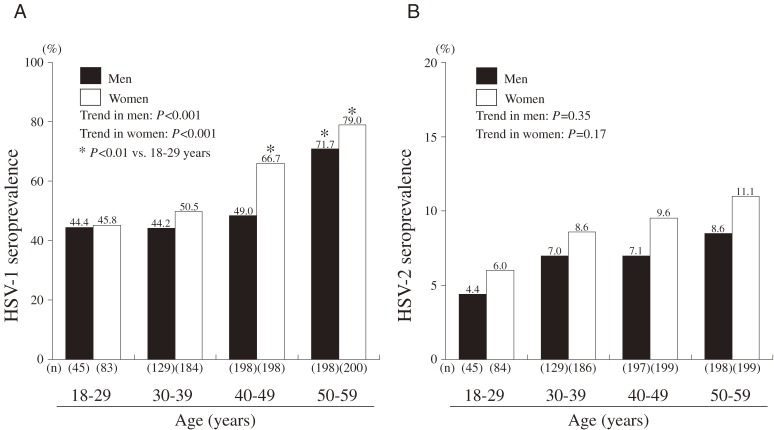
Seroprevalence of (A) HSV-1 and (B) HSV-2 by age and sex

The overall seroprevalences of HSV-1, HSV-2, and coinfection were 59.7%, 8.4%, and 3.7%, respectively. Seroprevalence was higher among women than among men for HSV-1 (63.3% vs. 55.4%, *P* = 0.16), HSV-2 (9.3% vs. 7.4%, *P* = 0.27), and coinfection (5.0% vs. 2.3%, *P* = 0.02), but only the difference in coinfection was statistically significant (Table 1). Figure 1A and 1B show HSV-1 and HSV-2 seroprevalence among participants by age and sex. In both sexes, the seroprevalence of HSV-1 and HSV-2 was lowest in participants aged 18 to 29 years and highest in those aged 50 to 59 years. In addition, seroprevalence was higher in women than in men, in all age brackets. There was an upward trend in HSV-1 and HSV-2 seroprevalence with advancing age in both sexes (*P* for trend < 0.001 in both sexes). In men, the prevalence of coinfection increased from 0% in those aged 18 to 29 years to 4.6% in those aged 50 to 59 years; a similar trend was observed in women: prevalence increased from 2.4% to 6.5%, respectively.

### Lifestyle-related risk factors for HSV-1 and HSV-2 infection

Age- and multivariate-adjusted ORs with 95% CIs for different risk factors of HSV-1 and HSV-2 infections are shown in Tables 2 and 3. Regarding lifestyle-related risk factors, alcohol drinking was associated with an increased risk for HSV-1 infection (OR: 1.81; 95% CI, 1.17–2.79 for current drinkers) in men. Current smokers had a higher risk of HSV-2 infection than never smokers among both men (OR: 4.13; 95% CI, 1.22–14.00) and women (OR: 2.26; 95% CI, 1.14–4.50).

**Table 2.** Age- and multivariate-adjusted logistic regression analysis for risk of HSV-1 infection in men and women in Hisayama, Japan

	Men						Women					
		
	Participants at risk	Positive for HSV-1	Age-adjusted OR (95% CI)	*P* value	Multivariate-adjusted OR (95% CI)	*P* value	Participants at risk	Positive for HSV-1	Age-adjusted OR (95% CI)	*P* value	Multivariate-adjusted OR (95% CI)	*P* value
Marital status												
​ Married	491	281	1 (referent)		1 (referent)		561	368	1 (referent)		1 (referent)	
​ Single	75	33	0.95 (0.55–1.63)	0.84	0.82 (0.47–1.43)	0.48	102	52	1.10 (0.67–1.80)	0.71	0.90 (0.54–1.50)	0.69
Smoking status												
​ Never	130	80	1 (referent)		1 (referent)		541	353	1 (referent)		1 (referent)	
​ Past	122	73	0.81 (0.49–1.37)	0.44	0.75 (0.44–1.28)	0.27	33	21	1.24 (0.58–2.64)	0.58	1.15 (0.53–2.47)	0.79
​ Current	318	163	0.70 (0.46–1.07)	0.10	0.66 (0.42–1.02)	0.06	91	47	0.75 (0.47–1.20)	0.23	0.72 (0.45–1.17)	0.19
Alcohol intake												
​ Never	119	53	1 (referent)		1 (referent)		363	229	1 (referent)		1 (referent)	
​ Past	23	16	2.37 (0.89–6.31)	0.08	2.41 (0.89–6.54)	0.08	24	14	0.81 (0.34–1.94)	0.64	0.88 (0.36–2.17)	0.78
​ Current	428	247	1.65 (1.09–2.51)	0.02	1.81 (1.17–2.79)	0.007	278	178	1.15 (0.82–1.61)	0.43	1.17 (0.83–1.65)	0.38

OR: odds ratio; CI: confidence interval; Multivariate adjustment was made for age, marital status, smoking status, and alcohol intake.

**Table 3.** Age- and multivariate-adjusted logistic regression analysis for risk of HSV-2 infection in men and women in Hisayama, Japan

	Men						Women					
		
	Participants at risk	Positive for HSV-2	Age-adjusted OR (95% CI)	*P* value	Multivariate-adjusted OR (95% CI)	*P* value	Participants at risk	Positive for HSV-2	Age-adjusted OR (95% CI)	*P* value	Multivariate-adjusted OR (95% CI)	*P* value
Marital status												
​ Married	490	39	1 (referent)		1 (referent)		562	56	1 (referent)		1 (referent)	
​ Single	75	3	0.56 (0.16–2.00)	0.37	0.60 (0.16–2.20)	0.44	104	6	0.66 (0.26–1.68)	0.39	0.70 (0.27–1.84)	0.47
Smoking status												
​ Never	129	3	1 (referent)		1 (referent)		541	44	1 (referent)		1 (referent)	
​ Past	123	9	3.13 (0.82–11.86)	0.09	3.03 (0.79–11.56)	0.11	34	4	1.76 (0.59–5.32)	0.31	1.71 (0.56–5.26)	0.34
​ Current	317	30	4.63 (1.39–15.52)	0.01	4.48 (1.33–15.04)	0.02	93	14	2.33 (1.20–5.54)	0.01	2.30 (1.16–4.54)	0.02
Alcohol intake												
​ Never	118	8	1 (referent)		1 (referent)		364	31	1 (referent)		1 (referent)	
​ Past	23	1	0.56 (0.07–4.75)	0.60	0.56 (0.07–4.81)	0.60	25	2	0.95 (0.21–4.21)	0.94	0.86 (0.19–3.89)	0.84
​ Current	428	33	1.12 (0.50–2.50)	0.78	1.01 (0.45–2.27)	0.99	279	29	1.29 (0.75–2.20)	0.35	1.16 (0.67–2.01)	0.59

OR: odds ratio; CI: confidence interval; Multivariate adjustment was made for age, marital status, smoking status, and alcohol intake.

### Risk of HSV-2 in the presence or absence of HSV-1 infection

Adjusting for other covariates, HSV-1 seropositive, as compared to HSV-1 seronegative, men and women had a lower risk of HSV-2 infection (for men: OR 0.30, 95% CI 0.15–0.61; for women: OR 0.56, 95% CI 0.33–0.97) (Table 4).

**Table 4.** The risk of HSV-2 in the presence or absence of HSV-1 infection by sex in men and women in Hisayama, Japan

	Men						Women					
		
	Participants at risk	Positive for HSV-2	Age-adjusted OR (95% CI)	*P* value	Multivariate-adjusted OR (95% CI)	*P* value	Participants at risk	Positive for HSV-2	Age-adjusted OR (95% CI)	*P* value	Multivariate-adjusted OR (95% CI)	*P* value
HSV-1												
​ Negative	254	29	1 (referent)		1 (referent)		244	29	1 (referent)		1 (referent)	
​ Positive	311	13	0.29 (0.14–0.58)	<0.001	0.30 (0.15–0.61)	<0.001	419	33	0.54 (0.31–0.94)	0.02	0.56 (0.33–0.97)	0.04

OR: odds ratio; CI: confidence interval; Multivariate adjustment was made for age, marital status, smoking status, and alcohol intake.

## DISCUSSION

We observed that in a general Japanese population the seroprevalence of HSV-1 and HSV-2 was 55.4% and 7.4% in men, and 63.3% and 9.3% in women, respectively. In age-stratified analysis, the seroprevalence of HSV-1 and HSV-2 increased significantly with age. Our study provides up-to-date data, and results that are valid with respect to sample size and sociodemographic correlates. We expect these findings to be useful for controlling HSV-1 and HSV-2 infection in the Japanese population.

Information on age- and sex-specific prevalence of HSV-1 and HSV-2 is essential to evaluate the burden of genital herpes and to optimize strategies to control it. It is also important because accumulating data indicate that HSV-2 infection may increase susceptibility to and transmission of human immunodeficiency virus.^14^ International comparison of HSV-1 and HSV-2 seroprevalences calculated using the same type-specific gG-capture ELISA method revealed that the seroprevalence of HSV-1 in Japan is similar to those reported in other industrialized countries (54%–77%).^2^^,^^15^^–^^19^ However, the prevalence of HSV-2 observed in our study was substantially lower than that in the United States (22%),^20^ but comparable with that reported in some European countries (4%–14%).^21^^–^^24^ In Japan, only 1 previous study reported the seroprevalences of HSV-1 and HSV-2: this 1993 study used a random sample of 105 men and 158 women from a rural area.^10^ This study also used a similar ELISA method for antibody assays and noted that the HSV-1 and HSV-2 seroprevalences were 54.4% and 1.8% in men and 59.6% and 1.2% in women, respectively. HSV-1 seroprevalence in this previous study was similar to that noted in the present study; however, HSV-2 seroprevalence was much lower in the previous study. There are several possible explanations for this discrepancy. First, the sample size of the previous study was small, and thus it is possible that the findings did not represent the actual seroprevalence of HSV-2. Second, the discrepancy might reflect differences in HSV-2 seroprevalence between regions. Third, HSV-2 seroprevalence might have increased in recent years. If seroprevalence has indeed increased, then an effective strategy is needed to control an epidemic of HSV-2 virus infection in Japan.

HSV-1 is most often transmitted via nonsexual contact, while HSV-2 infection is one of the most prevalent sexually transmitted infections worldwide. In our study, HSV-1 infection was widely prevalent, and its prevalence increased with age and peaked in the highest age bracket in both sexes. Although the prevalence of HSV-2 showed a similar age-related trend, the differences among age brackets were not statistically significant. An international review showed a similar tend in the rest of the world: the increase in age-associated seroprevalence is more pronounced for HSV-1 than for HSV-2.^2^ One reason for this phenomenon could be that HSV-2 prevalence is much lower than that of HSV-1, and that this lower HSV-2 prevalence results in limited statistical power to detect an age-prevalence relationship. Another plausible explanation is that diversity in sexual behavior among individuals may alter the age-related trend in HSV-2. The published literature from Japan shows that the prevalence of HSV-2 was 0% to 2% in blood donors, 7% to 17% in pregnant women, and 80% in commercial sex workers.^9^ From a public health perspective, the most effective means of HSV-2 prevention would be through appropriate sex education among the adolescent population.

In this study, the age-specific HSV-1 and HSV-2 seroprevalences were higher in women than in men. Such a trend has been observed elsewhere,^2^ and 2 studies have reported a similar trend for HSV-1.^16^^,^^25^ It has been postulated that the increased seroprevalence of HSV-2 in women is the result of more efficient male-to-female transmission of the virus, anatomical differences in susceptibility to infection, and/or the tendency of women to choose sex partners who are older than themselves.^26^^,^^27^

Studies investigating the interaction between the 2 infections have shown conflicting results, ie, controversy remains as to whether HSV-1 infection confers protection against HSV-2 infection.^28^ Some studies have shown a protective effect,^26^^,^^27^^,^^29^ while others have not.^30^^–^^32^ We found that HSV-2 was less prevalent among HSV-1-seropositive individuals than among those not infected with HSV-1. Our ability to determine whether HSV-1 infection was protective against HSV-2 infection was limited due to the cross-sectional nature of our study. However, because HSV-1 infection is generally acquired earlier in life than HSV-2, it seems reasonable to conclude that HSV-1 infection confers protection against HSV-2 infection.

In the present study, current alcohol drinking was associated with increased HSV-1 infection in men, while current smoking was associated with increased HSV-2 infection in both sexes. The increased risk for HSV-1 infection among alcohol drinkers is consistent with a study conducted in Europe that noted a marginally increased risk for HSV-1 infection.^33^ Although the exact pathway is unclear, immune suppression induced by drinking could be one explanation.^34^ Prospective studies are needed to confirm the relation between alcohol drinking and HSV-1 infection. Regarding smoking and HSV-2 infection, a cross-sectional study conducted in the United States^30^^,^^35^ and a multi-country survey^30^^,^^35^ both found that smoking is an independent predictor of HSV-2 infection. Although this association could be due to confounding by sexual behavior, immune suppression similar to that observed in HIV-positive individuals^36^ may play an important role. Moreover, smoking increases susceptibility to HSV-2 infection by damaging the cervical epithelium through DNA modification.^37^

The main strength of our study is its population-based design. Moreover, this is the first study with a sufficient number of participants to obtain reliable results regarding HSV-1 and HSV-2 seroprevalence in Japan. However, this study also has an important limitation. As most of the data were collected from sera stored in a repository established 5 years ago, we were unable to obtain information on sexual behavior or other related socioeconomic characteristics, including wage levels and educational background. Therefore, we could not examine the association between seroprevalence and these variables.

In summary, our study has produced reliable estimates of HSV-1 and HSV-2 seroprevalence, which will aid in assessing the burden of these infections in the general Japanese population. The present findings might also prove useful in formulating a set of multifaceted strategies to prevent and control HSV infection in Japan.
